# Cluster-based photography and modeling integrated method for an efficient measurement of cassava leaf area

**DOI:** 10.1371/journal.pone.0287293

**Published:** 2023-10-20

**Authors:** Jittrawan Thaiprasit, Porntip Chiewchankaset, Saowalak Kalapanulak, Treenut Saithong

**Affiliations:** 1 Center for Agricultural Systems Biology (CASB), Systems Biology and Bioinformatics Research Laboratory, Pilot Plant Development and Training Institute, King Mongkut’s University of Technology Thonburi, Bang Khun Thian, Bangkok, Thailand; 2 Bioinformatics and Systems Biology Program, School of Bioresources and Technology, King Mongkut’s University of Technology Thonburi, Bang Khun Thian, Bangkok, Thailand; University of the West Indies at Cave Hill, BARBADOS

## Abstract

Leaf area (*LA*) and biomass are important agronomic indicators of the growth and health of plants. Conventional methods for measuring the *LA* can be challenging, time-consuming, costly, and laborious, especially for a large-scale study. A hybrid approach of cluster-based photography and modeling was, thus, developed herein to improve practicality. To this end, data on cassava palmate leaves were collected under various conditions to cover a spectrum of viable leaf shapes and sizes. A total of 1,899 leaves from 3 cassava genotypes and 5 cultivation conditions were first assigned into clusters by size, based on their length (*L*) and width (*W*). Next, 111 representative leaves from all clusters were photographed, and data from image-processing were subsequently used for model development. The model based on the product of *L* and *W* outperformed the rest (*R*^*2*^ = 0.9566, *RMSE* = 20.00). The hybrid model was successfully used to estimate the *LA* of greenhouse-grown cassava as validation. This represents a breakthrough in the search for efficient, practical phenotyping tools for *LA* estimation, especially for large-scale experiments or remote fields with limited machinery.

## Introduction

Leaves are the largest proportion of the plant canopy and are gateways to biochemical processes, including photosynthetic light absorption, carbon uptake and assimilation, and transpiration [1]. Leaf area (*LA*) has been shown to be well correlated with the biomass yield of plants [2, 3], thus, it is typically employed to infer the growth and productivity of crop plants [3–6]. Together, *LA*, leaf biomass and number of leaves reflect the physiological response of plants to their environment, for example, responses to irrigation [7, 8], soil moisture, light, and heat [8–10], and also the competition among different forest plant species [11–13]. Information on *LA* is an essential data of decision support system for precise farming practices, such as the appropriate time for trimming, pruning, fertilization, and harvesting [14]. As an important basic property for assessing the physiology of plants, there is a need for effective *LA* measurement methods that provides high throughput data, yet being practical both in terms of time and labor requirements.

The measurement of *LA* is typically carried out by both destructive and non-destructive methods. Destructive (direct) methods entail detaching and measuring all individual leaves, for example, using grid paper, weighting paper, leaf area meter, whereas with non-destructive (indirect) methods, the *LA* measurement can be taken while leaves remain attached to plants [1, 15]. Destructive methods are generally more accurate, especially when more sample replicates are available, but are labor-intensive, costly, and quite literally devastating to plants, which make them impractical for large-scale experiments, where a huge number of samples need to be handled under time limitation, and also for real-time or continuous studies that monitor the sequential development of the plants.

Conversely, non-destructive methods are designed to minimize disturbances to plants, allowing the monitoring of leaf expansion during the course of development. Indirect methods introduce some levels of approximation and automation to save time, for example, photography-based approaches and non-photography-based approaches. Examples of the photography-based approach estimate *LA* from processed leaf images taken by scanners, digital cameras, or a hand-held *LA* meter (LI-3000, LI-COR). Mathematical models have frequently been used for *LA* estimation to save time and cost, and cope with irregular leaf shapes and sizes, mostly relying on the simple linear regression equation on agronomic leaf traits [13, 16–22], or multiple stepwise regression [20]. These models are constructed based upon the relationship between morphological parameters of leaves and the measured *LA*. Accuracy of the approximation, hence, depends on how the parameters can precisely describe complex shapes of plant leaves, while inclusively taking into account their natural variation under the studied condition. Montgomery [13] proposed the first equation for *LA* prediction in corn using regression analysis: *LA* = a*L*W where *a* represents the coefficient of correlation between *LA* and the product of leaf length (*L*) and leaf width (*W*). Ever since, mathematical models for *LA* estimation have been developed for various plant species with simple or compound leaves, including green bean [17], faba bean [23], saffron [24], Persian walnut [3], rose [20], and citrus [25]. Key parameters in the models include morphological descriptors of leaves such as leaf length, leaf width, and petiole length [18, 20, 26]. The empirical function for *LA* estimation can be written as *LA = f(p*_*i*_*)*, where *p*_*i*_ is the morphological parameter(s). Incorporating these mathematical equations in remote sensing technology would assist rapid plant monitoring and accurate information generation for decision-making systems in precision farming technology [27].

There are only a few existing *LA* models for palmate compound leaves [19, 22, 28], the characteristic leaf morphology found in economic crops such as marijuana, maples, horse chestnuts, buckeyes, and cassava. Generally, plant morphology has been described based on geometrical descriptor (s), including forms and architecture. In this study, we also used simple geometric measures such as length (*L*) and width (*W*) to explore the variations in cassava leaf morphology. Palmate compound leaves of cassava consist of leaflets attached to a common point at the distal end of the petiole, and the size and dry matter content are important yield indicators [4, 29, 30]. A high degree of sophistication and specificity is crucial for models to cope with the complex, irregular forms, and variation of the palmate leaves. The latest *LA* model of cassava was developed from 140 leaves of the IAC 576–70 cultivar based on a simple linear regression of the length, width of the central lobe, and number of lobes [22]. It requires model parameters that are too detailed, time-consuming, and often logistically demanding to collect in field conditions. Moreover, identifying the central lobe can be tricky in some cultivars with even lobes. Herein, we developed an alternative model using simple morphological descriptors, which were formulated from a wider range of data, 3 cultivars and 5 cultivation conditions. Our model, thus, was expected to handle the cultivar-based variations in leaf morphology in different growth conditions well. Furthermore, we combined the mathematical model estimator with cluster-based photography and proposed an effective hybrid *LA* measurement method that makes conducting large-scale morphological studies with relatively easy to attain.

## Materials and methods

### Plant materials

Cassava (*Manihot esculenta* Crantz) leaves were collected from three morphologically distinct cassava cultivars: Kasetsart 50 (KU50), Hanatee (HN), and Rayong 9 (R9) grown in five different experimental conditions, which comprised combinations of two cultivations (field and greenhouse) and watering (irrigated and rainfed) systems: field-irrigated KU50, field-irrigated R9, field-rainfed R9, greenhouse-irrigated KU50, and greenhouse-irrigated HN (Table 1). In the field, KU50 and R9 were propagated from stem cuttings under irrigated and rain-fed conditions at KhonKaen University, Thailand. All fully-expanded leaves were harvested at 8–12 months after planting (MAP) to represent the maximum range of leaf size as possible. All plants grown in a field were randomly selected 1–2 of 20 plants per plot. Each plot was grown with separate species and age. The distance between the plants and the row was 0.8 m × 1 m. In the greenhouse, leaves of KU50 and HN were harvested at 2 MAP at Forschungszentrum Jülich, Germany [31]. In total, 1,899 fully-expanded leaves were collected from 15 plants grown in the field and greenhouse (Table 1).

**Table 1:** Five experimental conditions of cassava leaves used in this study.

Experimental conditions	Cultivation conditions		Cultivars	Ages (MAP)	Number of plants	Number of leaves
	Cultivation systems	Irrigation systems				
1	field	irrigation	KU50	8,12	5	973
2	field	irrigation	R9	8,12	2	513
3	field	rainfed	R9	8,12	2	335
4	green house	irrigation	KU50	2	3	58
5	green house	irrigation	HN	2	3	20
** **				**Total**	**15**	**1,899**

### Measurement of leaf area and morphological descriptors

After harvesting, all fresh leaves were immediately processed for measurement to avoid wilting-associated error. Following the ImageJ-based method [32], leaves were placed on a white background chart with a ruler scale and were flattened using a transparent acrylic plate which was placed over them to prevent shadow interference during imaging. Photographs were taken by a digital camera (Canon PowerShot G1X with effective resolution of 14.3 megapixels, Canon) at a perpendicular position to the axis (S1A Fig) to minimize perspective distortion. Determination of *LA* (cm^2^) from these images was carried out using ImageJ software [33]. In addition, applying the LA meter-based method, the individual and total *LA* of fresh leaves were measured using the LI-3100c Leaf Area Meter, LI-COR^®^

**Fig 1:**
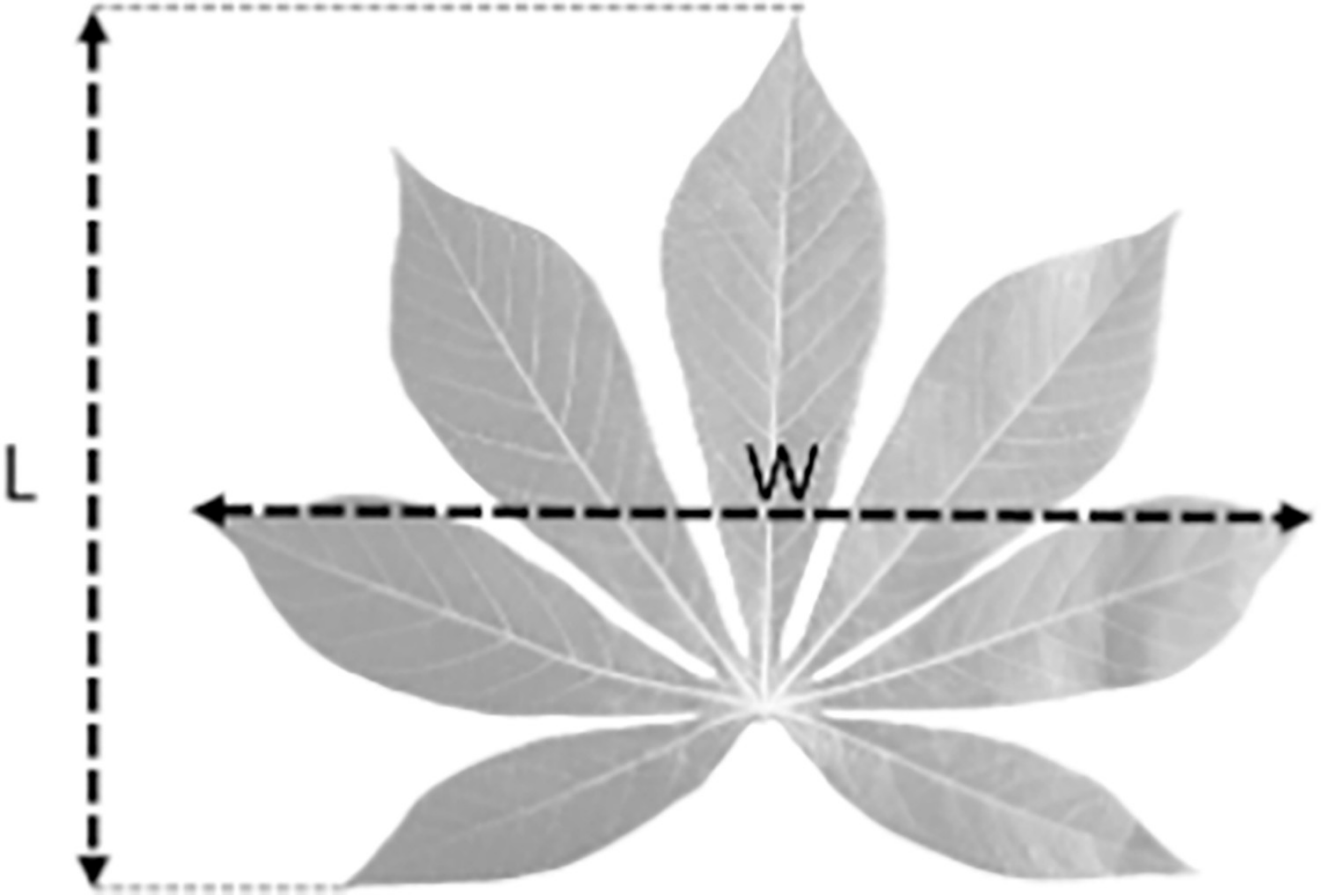
Diagram of cassava palmate leaf with width (*W*) and length (*L*) segments defined.

The dimension parameters of cassava leaves were determined for representing simple morphological descriptors of fully-expanded leaves for the *LA* estimator-model development. The dimension parameters, including the width (*W*)—distance between the apex of the farthest protruding leaflets on each side of the central leaflet, and the length (*L*)—distance between the apex of the central leaflet and apex of the southernmost leaflet (Fig 1), were determined by the ImageJ approach (S1B Fig).

### Leaf area estimator model development

#### Cluster-based model

**Fig 2:**
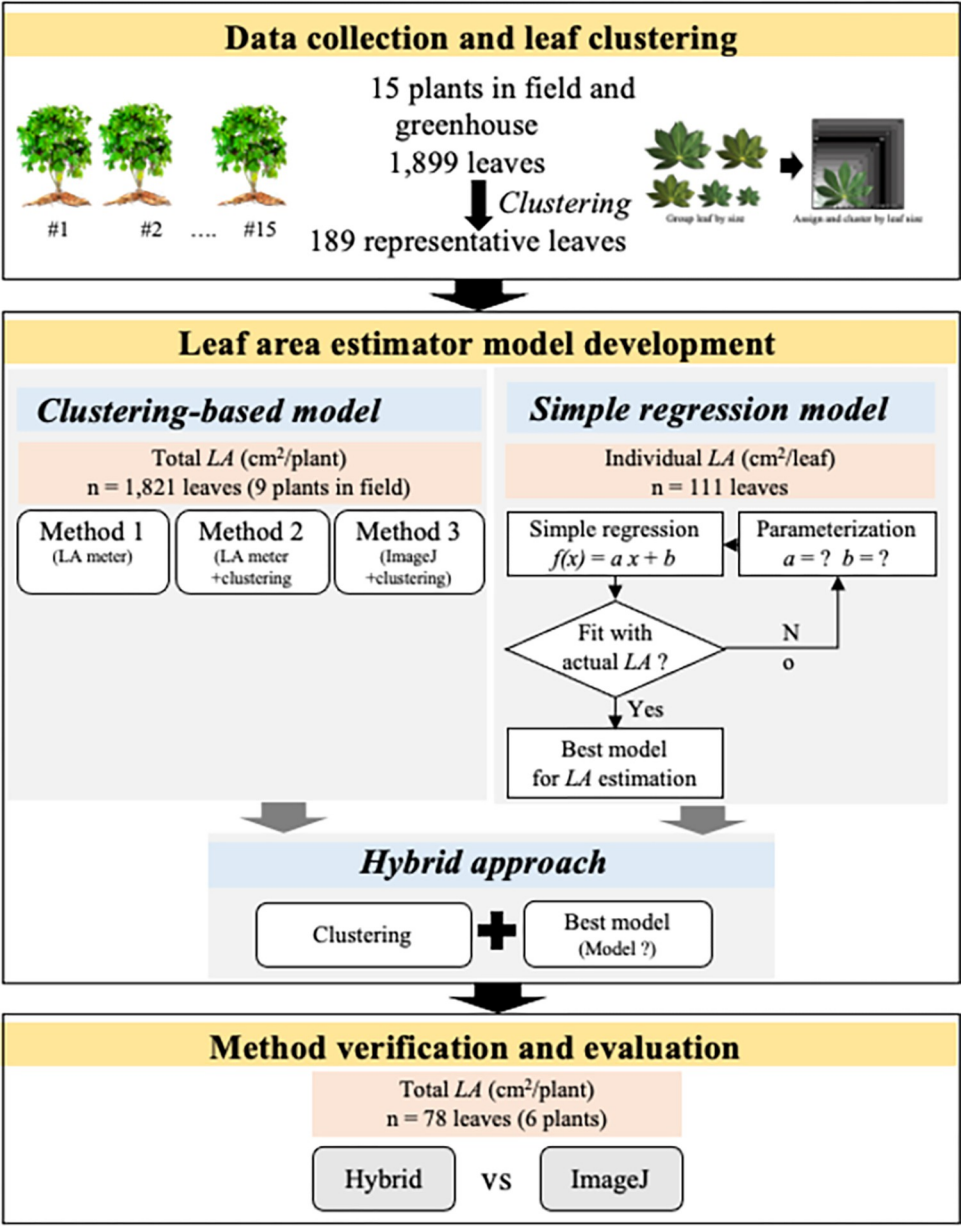
Framework of leaf area estimator model development.

To reduce the number of leaves to be measured, all leaves were clustered using a scoring board developed from scalable squares of width by length (S2A Fig). The range of the scales, spanning from 3.5 x 3.5 to 15.5 x 15.5 square inch, at an incremental rate of 1x1 square inch, was designed according to the varietal differences in size observed. Here, *L* refers to numbers along the vertical or diagonal axis (y-axis on scoring board)–depending on leaf orientation, and *W* refers to numbers on the corresponding perpendicular axis (x-axis on scoring board). In brief, leaves of each plant were grouped by size and assigned to clusters based on the *L* x *W* dimension of the scoring board (S2B Fig). For each cluster, at least a couple representative leaves were randomly sampled for more precise *LA* measurements using ImageJ. The total *LA* per plant was finally calculated based on Eq 1

$$Total\;LA\;per\;plant=\sum_{i=1}^{N}({LA}_{rep}*{LN)}_{i}$$ (1)

where *i* is the cluster number of the total *N* clusters in a plant, *LA*_*rep*_ is *LA* of the representative leaf from the *i*-th cluster, and *LN* is the number of leaves in the *i*-th cluster. The total *LA* per plant obtained by this approach was subsequently compared with values from the LA-meter approach (Fig 2).

#### Simple regression model

The simple linear regression model of *LA* was developed based on the leaf descriptors, *L* and *W*, and the overall process is depicted in Fig 2. The relationship of the three determinants, *LA*, *W*, and *L* was inferred from 111 representative cassava leaves. The detail of selected 111 leaves was described in S3 File. Particularly, the regression of *LA* by *L*, *W*, *L*+*W*, L*W, and *L*/*W* was examined. The model performance was evaluated according to correlation coefficient (*R*^*2*^) and root mean square error (*RMSE*) values. The best model with the highest *R*^*2*^ and lowest *RMSE* [34] was selected.

#### Hybrid model

The hybrid approach incorporates both the cluster-based photography and regression approaches described earlier and is intended for use in non-destructive estimation of total *LA*. In brief, leaves were assigned to size-based clusters using a scoring board. Then, the *LA* of leaves in each cluster was estimated using the best regression model. The total *LA* per plant was estimated using Eq 1. To analyze its reliability, comparisons with the cluster-based photography method were made based on the *RMSE*, *MAE*, and *MRE*. Seventy-eight leaves from three KU50 and three HN cultivars (6 plants in total) grown in greenhouse conditions were used for this experiment.

### Model evaluation and verification

Performance of the best fit model developed from 111 leaves was evaluated through *LA* estimation of cassava leaves collected from independent studies. The correspondence of the model-estimated *LA* (X) and measured values (Y) were demonstrated by the x-y plot and coefficient of determination (*R*^*2*^) of the linear regression. In addition, the accuracy of model estimation was examined by multiple statistical measures of residual errors, including the root mean square error (*RMSE;* Eq 2), the mean absolute error (*MAE;* Eq 3), and the mean relative absolute error (*MRE;* Eq 4), following Hyndman and Koehler [35]. The errors were defined as the distance between *LA* estimated (*M*_*o*_) and *LA* measured by the benchmark method (*M*_*b*_), which herein corresponded to the model estimated *LA* and measured *LA*, respectively.


$$RMSE=\sqrt{\frac{1}{n}\sum_{i=1}^{n}{\left({LA}_{M_{0}}-{LA}_{M_{b}}\right)}^{2}}$$ (2)

$$MAE=\frac{1}{n}\sum_{i=1}^{n}\left|{LA}_{M_{0}}-{LA}_{M_{b}}\right|$$ (3)

$$MRE=\frac{1}{n}\sum_{i=1}^{n}\frac{\left|{LA}_{M_{0}}-{LA}_{M_{b}}\right|}{{LA}_{M_{b}}}$$ (4)

## Results and discussion

### Variation in cassava leaf morphology

Cassava leaves are large, palmate-shaped, and contain three to seven lobes attached to a long petiole. The leaf size can vary depending on the cultivar, leaf position on a plant (i.e. top and bottom), age, cultivation system, and stress condition being exposed [27, 36]. For instance, KU50 and R9, two popular Thai cassava cultivars for the starch industry, have thinner leaf lobes than HN, another Thai cultivar. Generally, leaves tend to grow smaller in greenhouses than in field conditions. This variability in leaf shapes and sizes could affect the *LA* estimation model; thus, different leaf morphological types were captured in this study. A total of 1,899 cassava leaves from 15 plants of different ages and genotypes growing in 5 different cultivation systems as mentioned in Table 1.

Results of 189 representative leaves from the 15 plants (Table 2) revealed age-related differences in the leaf area of cassava mature leaves of the same cultivar. For example, leaves of R9 at 8 MAP during late-stage of vegetative growth (Plant no. 6 and 8 in Table 2) were larger than R9 leaves at 12 MAP of the end of vegetative growth (Plant no. 7 and 9 in Table 2).

**Table 2:** Variations in morphological descriptors, length (*L*) and width (*W*), and leaf area (*LA*) of 189 representative cassava leaves measured using the ImageJ-based method.

No. of plant	Sample description*		Number of representative leaves	Length (*L*), cm				Width (*W*), cm				*LA* by ImageJ-based method (cm^2^)			
	cultivars	conditions		Min	Mean	Max	SD	Min	Mean	Max	SD	Min	Mean	Max	SD
#1	KU50	FL, I, 8 MAP	13	10.47	21.56	29.26	5.14	13.08	29.01	40.58	7.55	46.22	256.68	514.89	124.30
#2	KU50	FL, I, 12 MAP	17	8.14	16.37	23.93	4.48	10.65	22.89	34.63	6.04	24.45	154.47	333.65	83.12
#3	KU50	FL, I, 12 MAP	16	7.94	13.35	20.96	3.75	12.40	19.84	28.70	5.00	35.37	109.17	278.58	71.36
#4	KU50	FL, I, 12 MAP	15	8.18	15.14	19.33	3.59	11.09	19.99	27.22	5.32	42.34	128.50	247.18	63.05
#5	KU50	FL, I, 12 MAP	9	15.89	20.47	26.59	3.56	20.81	28.21	35.10	4.98	126.66	250.90	398.49	97.12
#6	R9	FL, I, 8 MAP	7	20.87	23.97	26.81	2.21	25.76	30.66	35.54	3.24	248.67	341.63	459.55	71.32
#7	R9	FL, I, 12 MAP	12	10.56	16.59	26.59	3.36	13.91	22.12	35.10	4.57	59.22	162.06	398.49	63.08
#8	R9	FL, R, 8 MAP	10	18.34	23.08	27.52	3.38	23.09	29.96	37.76	5.21	198.00	323.97	489.05	97.69
#9	R9	FL, R, 12 MAP	12	11.53	17.16	22.08	3.28	15.37	23.16	30.55	4.87	71.95	169.83	253.17	61.81
#10	KU50	GH, I, 2 MAP	5	8.57	10.79	12.74	1.50	10.19	13.76	16.29	2.24	40.12	64.36	80.45	15.20
#11	KU50	GH, I, 2 MAP	7	9.89	12.25	13.33	1.19	10.51	15.95	17.71	2.62	44.57	88.16	116.48	22.54
#12	KU50	GH, I, 2 MAP	8	10.91	12.53	13.85	1.07	14.17	17.19	18.96	1.66	72.49	98.79	122.40	18.47
#13	HN	GH, I, 2 MAP	20	7.22	14.97	18.91	3.63	5.00	21.49	29.21	6.83	11.73	135.02	210.40	59.67
#14	HN	GH, I, 2 MAP	20	7.01	15.33	18.44	2.55	8.77	23.26	28.17	4.56	25.48	143.26	189.69	38.60
#15	HN	GH, I, 2 MAP	18	6.28	15.47	19.93	3.87	10.23	22.03	28.66	5.98	26.82	143.67	213.31	60.03
	Total		189	-	-	-	-	-	-	-	-	-	-	-	-
	Minimum		5	6.28	10.79	12.74	-	5.00	13.76	16.29	-	11.73	67.36	80.45	-
	Maximum		20	20.87	23.97	29.26	-	25.76	30.66	40.58	-	248.67	341.63	514.89	-
	Average		12.6	10.79	16.60	21.35	3.10	13.67	22.63	29.61	4.71	71.61	171.36	287.05	63.16
	*CV*		38.97%	28.64%				28.61%				58.03%			

*KU50 = Kasetsart50, R9 = Rayong9, and HN = Hanatee; FL = field; GH = greenhouse; I = irrigated; R = rainfed; MAP = Month after planting.

The variation in leaf morphology was evident from the range of values obtained for the descriptors, *L* with a 6.28–29.26 cm range and SD of 3.10, and *W* with a 5.00–40.58 cm range and SD of 4.71, indicating higher variability. The *LA* ranged between 24.45 and 514.89 cm^2^ at 8–12 MAP in field conditions (Plant no. 1–9) and from 11.73 to 213.31 cm^2^ for plants at 2 MAP in greenhouse conditions (Plant no. 10–15) with a total *LA* average of 171.36 cm^2^. The high standard deviation of *LA* (Maximum SD of 124.30) showed a wide variation in leaf sizes used for model development. In comparison, coefficients of variation (*CV*) of the three variables, *L*, *W*, and *LA*, were 28.64%, 28.61%, and 58.03%, respectively, showing high dispersion of *LA* (more than 50%) and comparable distribution of *W* and *L*. The high dispersion of *LA* indicated that this dataset comprises a wide variety of cassava leaves as required for *LA* estimation model development.

**Fig 3:**
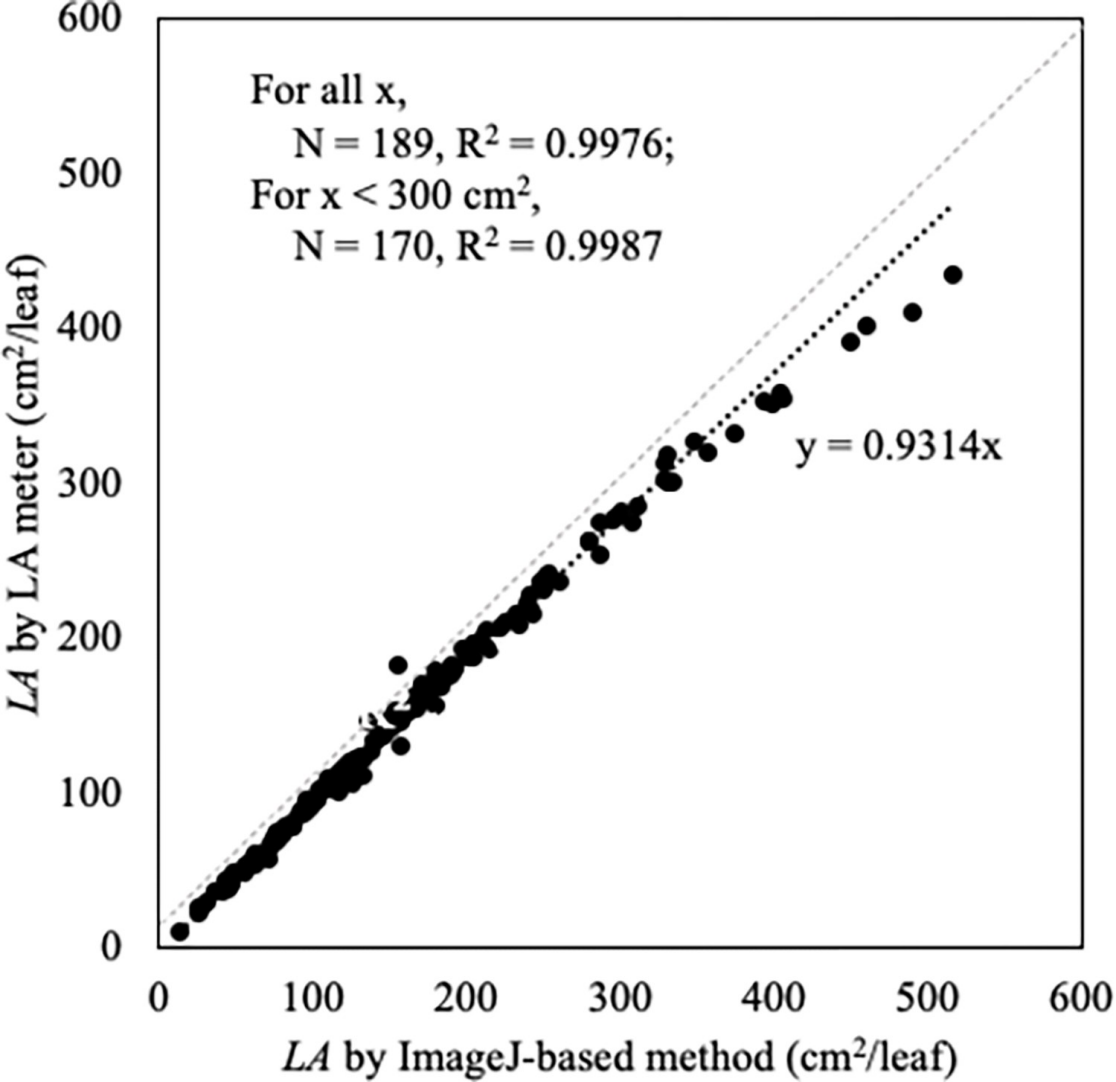
Comparison of *LA* estimated by the ImageJ-based method (*N* = 189) with the standard leaf area meter (LI-200 Leaf Area Meter, LI-COR^®^).

As to ensure the accuracy of the *LA* estimation by ImageJ-based measurement (Table 2), which was employed here according to its practicality to the experiment, we investigated the resulting values against that from the standard LA-meter (LI-3100c Leaf area meter, LI-COR^®^) approach. The comparison was based on 189 sampled leaves (Table 1). The results showed that both methods gave comparable *LA* values (*R*^*2*^ = 0.9976), especially for *LA* values below 300 cm^2^ (Fig 3). The noticeable variation in *LA* values observed for *LA* above 300 cm^2^ could be caused by the under-representation of *LA* of larger leaves due to the limited sample detecting area of leaf area meter, sample width area of 25 cm wide. The regression coefficient showed strong correlation between *LA* obtained from both methods (For all *x*, *R*^*2*^ = 0.9976, *N* = 189; for *x* < 300 cm^2^, *R*^*2*^ = 0.9987, *N* = 170; where *x* is *LA* calculated by ImageJ-based method) with a slope of 0.9314 (Fig 3). These results are corresponding to those reported for cut rose [20], tomato, and corn [37], indicating the reliability of the ImageJ-based method.

### Improving practicality of *LA* estimation by clustering

The total leaf area is always required to represent shoot growth during plant development as well as shoot phenotype characteristics of plants. To shorten experimental time and make phenotyping more practical, the image-based method was improved by adding a clustering step (S2 Fig). To analyze the reliability of an approach, the total *LA* per plant calculated by the improved method (Method 1 in Fig 2, M1) was compared to values from the LA meter-based method *with* clustering (Method 2 in Fig 2, M2) and the LA meter-based method *without* clustering (Method 3 in Fig 2, M3) using *RMSE*, *MAE*, and *MRE*. This experiment was carried out using 1,821 cassava leaves obtained from nine field-grown plants—five KU50, two R9-irrigated, and two R9-rainfed replicates (Table 3).

**Table 3:** Total leaf area of field-grown cassava plants calculated by three different methods: ImageJ-based method with clustering (M1), LA meter-based method *with* clustering (M2), and LA meter-based method *without* clustering (M3).

No. of plant	Sample		Number of leaves	Number of representative leaves	Total LA per plant (m^2^/plant)			Residuals		
	cultivars	conditions			M1	M2	M3	|M1—M2|	|M1—M3|	|M2—M3|
#1	KU50	FL,I	209	13	51.93	48.73	51.03	3.20	0.90	2.30
#2	KU50	FL,I	397	17	65.28	62.05	60.56	3.23	4.73	1.49
#3	KU50	FL,I	198	16	23.50	22.20	21.06	1.29	2.44	1.15
#4	KU50	FL,I	113	15	16.36	15.74	13.12	0.62	3.24	2.61
#5	KU50	FL,I	56	9	13.77	12.59	10.82	1.18	2.95	1.77
#6	R9	FL,I	377	7	62.22	56.79	35.74	5.44	26.48	21.05
#7	R9	FL,I	136	12	27.27	25.75	23.92	1.52	3.36	1.84
#8	R9	FL,R	208	10	64.59	60.75	60.19	3.84	4.40	0.56
#9	R9	FL,R	127	12	23.09	21.89	19.39	1.20	3.69	2.50
						***RMSE***		2.83	9.39	7.24
						***MAE***		2.39	5.80	3.92
						***MRE***		0.06	0.21	0.14

The results revealed that the total *LA* per plant for both methods *with* clustering (M1 and M2) were not significantly different (Table 3 (*RMSE* = 2.83, *MAE* = 2.39, *MRE* = 0.06), and S4 Fig). The total *LA* from M3 (*without* clustering) was quite different from M1 and M2 (*MRE* of 0.21 and 0.14, respectively). The difference was manipulated by the high residual of 26.48, from the cassava plant no. 6, which had more number of large leaves than others (Table 3). Excluding the cassava plant no. 6, the residues *MAE* between the 3 methods; M1vs M2, M1vs M3, and M2 vs M3, reduced to 1.79, 2.86 and 1.58, respectively. The result indicated a gap of improvement for the clustering-based method (M1 and M2) through optimizing the *LA* measurement for large-size leaves, or alternatively minimizing the sensitivity to datasets with high error magnitude, such that happened with the plant no. 6. In summary, the clustering was demonstrated to effectively reduce the sample load in estimation of *LA*, and enhanced practicality of the image-based method to handle large-scale measurement.

### Regression model for cassava *LA* estimation

**Fig 4:**
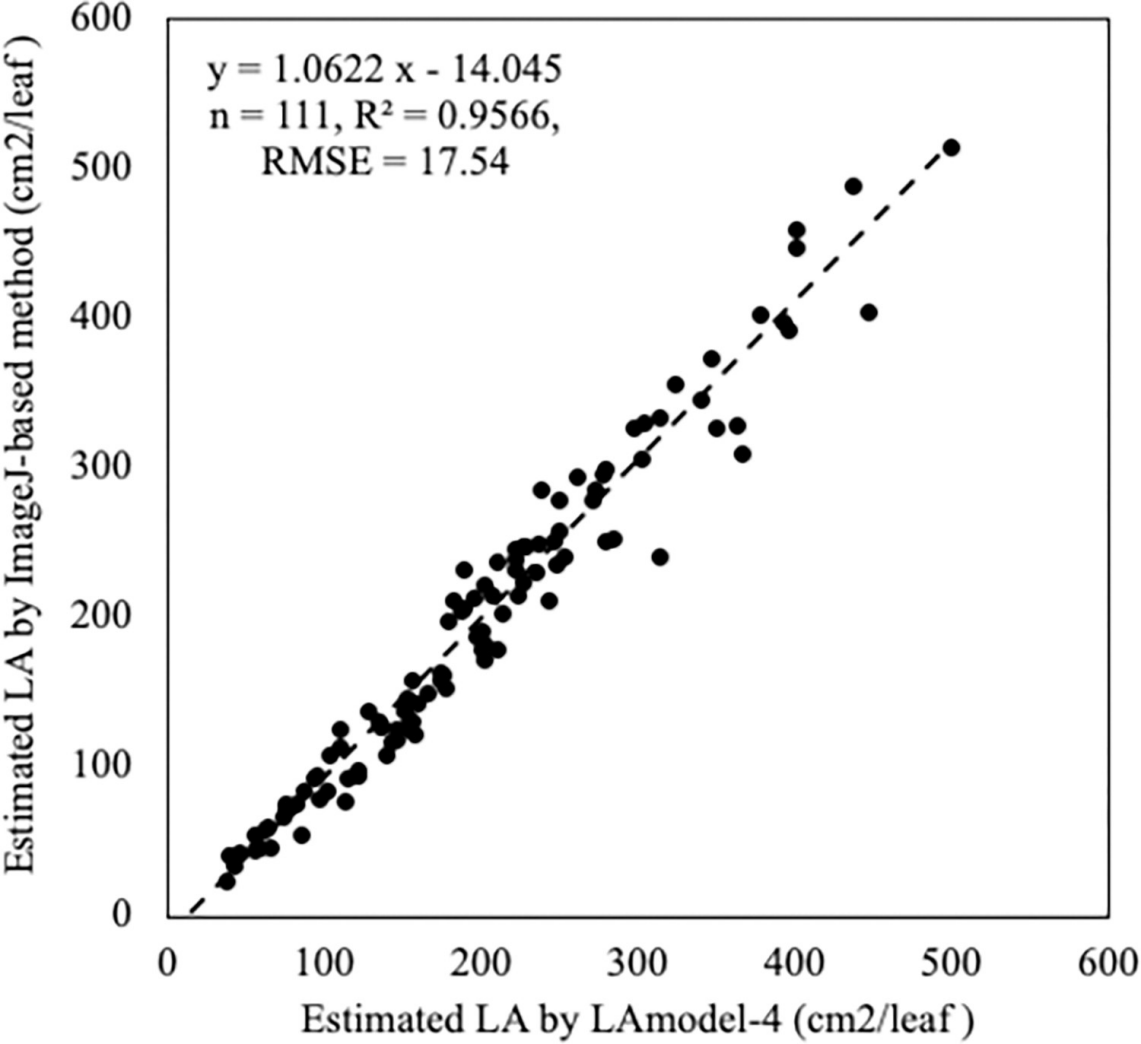
Comparison of leaf area estimation by the LA_Model-4_ and ImageJ-based method.

Since the *LA* estimation using the ImageJ-based method still contained a time-consuming step, a mathematical model was incorporated to further mitigate the tedious process. Five linear regression models were developed based on the morphological descriptors, namely *L*, *W*, *L*+*W*, L*W, and *L*/*W*. The coefficient of determination (*R*^*2*^) in regression analysis showed that the estimated *LA* by these models, except for the *L/W* model, well corresponded to the experimental measurements, with *R*^*2*^ values of 0.92, 0.90, 0.93, 0.96, and 0.02, respectively (Table 4, S2 Fig). Accordingly, the *L/W* model was left out, and the rest were used for the regression model development. The *L*, *W*, *L*+*W*, and L*W values of the representative leaves (111) were fitted separately and together to the linear regression equation: *f(x)* = *a* * (*x*), where *f(x)* represents the *LA*, *x* represents the model of leaf morphological descriptors, and *a* is the model constant defined by the slope of the linear regression model. Table 4 shows the estimated model parameters of the selected variables. The L*W model (LA_Model-4_) showed the best fit with the measured *LA* from the photography method with the highest *R*^*2*^ of 0.9566, and *RMSE* = 17.54 cm^2^ (Table 4). The *LA*_Model-4_ model is based on the area of rectangles, which has been widely used in studies of various foliage species, including corn [13, 38], green bean [17], cucumber [39], grasses and legumes [3, 40]. The comparison of *LA* estimation by LA_Model-4_ and the ImageJ-based method showed a strong correlation with *R*^*2*^ = 0.9566, and slope = 1.0622 (Fig 4). Thus, the model predicted *LA* values with high accuracy.

**Table 4:** Regression models of palmate leaf area estimation.

Model name	Independent variable (*x*)	*R*^*2*^ of actual *LA* vs *x*	Regression equation (*LA* = a*(*x*))	*R*^*2*^ of measured *LA* vs *x*	RMSE
*LA*_Model-1_:	*L*	0.92	*f(x)* = 11.58*(*x*)	0.7221	44.30
*LA*_Model-2_:	*W*	0.90	*f(x)* = 8.54*(*x*)	0.6921	60.58
*LA*_Model-3_:	*L+W*	0.93	*f(x)* = 4.92*(*x*)	0.7113	155.47
*LA*_Model-4_:	L*W	0.96	*f(x)* = 0.42*(*x*)	0.9566	17.54
*LA*_Model-5_:	*L/W*	0.02^ϯ^	-	-	

^ϯ^ This value is not significant at *p-*value ≤ 0.01

The previous mathematical models for *LA* estimation by Karim et al. (2010) and Zanetti et al. (2017) were based on the morphotype Philippine and cultivar LAC 576–70, respectively. The top-ranked model in Karim et al. relied on the product of mean of leaf-lobe length and width, with *R*^*2*^ of 0.97 (n = 100), whereas the best model in Zanetti et al. was based only on length of the central lobe, with *R*^*2*^ of 0.92 (n = 140). The *R*^*2*^ of our model is comparable with theirs. However, their morphological descriptors, mean of leaf-lobe length and length of the central lobe, are too detailed and might be daunting to execute, particularly in huge experimental setups.

### A hybrid approach: Performance, practical guideline, and extension

In this study, we proposed a hybrid approach for *LA* estimation, which is a combination of the proposed linear regression model and the clustering method. To analyze its reliability, comparisons with the cluster-based photography method were made based on the *RMSE*, *MAE*, and *MRE*. Seventy-eight leaves from three KU50 and three HN cultivars (6 plants in total) grown in greenhouse conditions were used for this experiment. The detail of plant selection for model testing was described in S3 File.

The difference between the *MRE* of (1) the hybrid approach and (2) the ImageJ-based method was 6.0%. The total *LA* estimated by the proposed non-destructive method (1) showed higher residuals when the total leaf area was larger (plant #13 - #15 in Table 5). The observed results may reflect the cumulative error of the estimation from each individual leaf sample. However, the proposed non-destructive method (1) was highly correlated with and the original destructive method (2) with *r* = 0.9996, indicating its reliability.

**Table 5:** Comparison of total *LA* per plant obtained by the hybrid approach (1), and ImageJ-based method (2).

No. of plants	Sample		Number of leaves	Total *LA* per plant (m^2^/plant)		Residuals
	cultivars	conditions		Hybrid method (1)	ImageJ-based method (2)	|(1)—(2)|
#10	KU50	GH,I	5	0.31	0.32	0.01
#11	KU50	GH,I	7	0.58	0.62	0.04
#12	KU50	GH,I	8	0.72	0.79	0.07
#13	HN	GH,I	20	2.90	2.70	0.20
#14	HN	GH,I	20	3.09	2.87	0.22
#15	HN	GH,I	18	2.74	2.59	0.15
					***RMSE***	**0.14**
					***MAE***	**0.11**
					***MRE***	**0.06**
					***r***	**0.9996**

**Fig 5:**
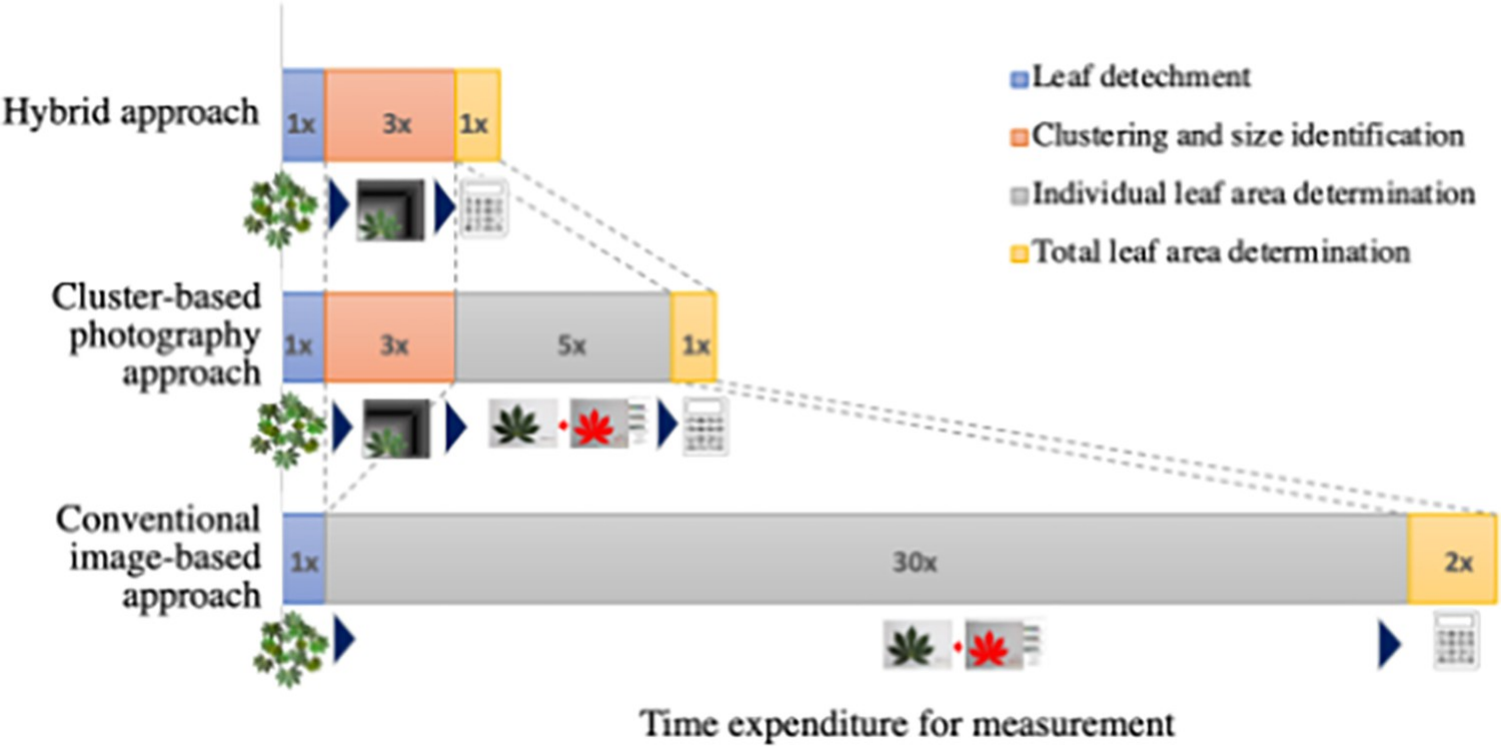
Schematic workflow of hybrid approach compared time expenditure with 3 methods of *LA* estimation for large-scale experiments, (1) conventional image-based approach (2) cluster-based photography approach, and (3) hybrid approach (Cluster-based photography and modeling integrated method). (Note 1x estimate 1 hour/10 plants).

The non-destructive hybrid approach was proposed to expedite the *LA* estimation in large-scale studies, based on our knowledge of the morphological characteristics, while ensuring high prediction accuracy. The approach substantially reduces the workload both in terms of quantity of leaves to be analyzed and the duration for *LA* determination, making it particularly suited for large-scale studies (Fig 5). In this study, we provide S1 File comprising two calculation table and one datasheet for supporting our hybrid approach (S1 File; Sheet S1.1), regression model for different palmate leaves (S1 File; Sheet S1.2), and record paper used on-field (S1 File; Sheet S1.3), respectively. In brief of hybrid approach (detail of implementation was in S3 File), leaves are grouped by a scoring board (S2 File). The value of *W*, *L*, and amount of leaf in each cluster are recorded on printout of datasheet (S1 File; Sheet S1.3) and filled on Sheet S1.1 of S1 File. The area of each representative leave was estimated by regression model-4 (LA_Model-4_) or 0.42 * *L* * *W*. Finally, the calculation table of Sheet S1.1 (S1 File) will provide the total *LA* per plant by the summation of individual *LA* * *LN* per plant (Fig 5). While our method was believed to accommodate *LA* estimation in variety of leaf morphology, the current version may provide superior results with more accuracy in lanceolate-based leaves (KU50, R9, and HN). In case of the morphology of palmate leaves differing from our cultivars; KU50, R9, and HN, our proposed method by design is easy to adapt and improve precision for all kinds of leaf morphology using the tools provided in S1 File; Sheet S1.2. Another advantage of this hybrid approach for palmate leaves is that it allows for continuous monitoring of leaves through the development stages until harvest.

## Conclusions

Leaf area is an important agronomic indicator of plant growth and health. Nevertheless, measurement of *LA* by existing methods can be time-consuming, laborious, and expensive, especially for large-scale studies. Thus, *LA* estimation models are needed for accurate and rapid real-time monitoring of responses of plants to short and long-term changes in the environment, crucial for yield optimization. Existing mathematical models for *LA* estimation are sensitive to leaf types and are not suited for the palmate leaves of cassava, even though they are based on basic agronomic parameters as the length (*L*) and width (*W*) of leaves. In this study, a simple regression model for estimating the *LA* of cassava was developed using palmate leaves of different genotypes grown under different conditions. The *L* and *W* were used as independent variables for the *LA* estimation model development. The linear regression model based on the product of *L* and *W* (L*W) proved the best for predicting *LA* with high accuracy (*R*^*2*^ = 0.9566, and *RMSE* = 17.54). The ImageJ-based method integrated with cluster-based photography, despite reducing the workload and expediting the *LA* estimation process, showed sensitivity to datasets with high error magnitude. The hybrid approach, which is a combination of the regression modeling and the clustering approaches, proved highly reliable and promising for estimating the *LA* of cassava.

## Supporting information

S1 Fig Measurement of morphological descriptors, a) LA, b) L, and c) W, by the ImageJ-based method. (TIFF)

S2 Fig Cluster-based photography method a) Developed clustering board used to define the approximate L and W (cm) of representative leaves, and b) Workflow of the cluster-based photography method. The procedure starts with leaf grouping, assigning leaf clusters using clustering board, taking images of representative leaves, and determining leaf area by ImageJ program.
(TIFF)

S3 Fig Comparisons of the LA data from the ImageJ-based method and the LA estimated by the four regression models. (TIFF)

S4 Fig Box plots of total LA per plant obtained from 3 different *LA* methods: ImageJ-based method with clustering (M1), LA meter-based method *with* clustering (M2), and LA meter-based method *without* clustering (M3). ns is not significant difference at α = 0.05.
(TIFF)

S1 File Calculation table for palmate leaf area estimation. (XLSX)

S2 File Clustering board for grouping palmate leaf of cluster-based photography method. (PDF)

S3 File Supplementary information of plant materials. (DOCX)
