# Supplementary figures and images for: Cluster-based photography and modeling integrated method for an efficient measurement of cassava leaf area
Source: PLoS One. 2023 Oct 20;18(10):e0287293. doi: 10.1371/journal.pone.0287293 (PMC10588870; doi:10.1371/journal.pone.0287293)

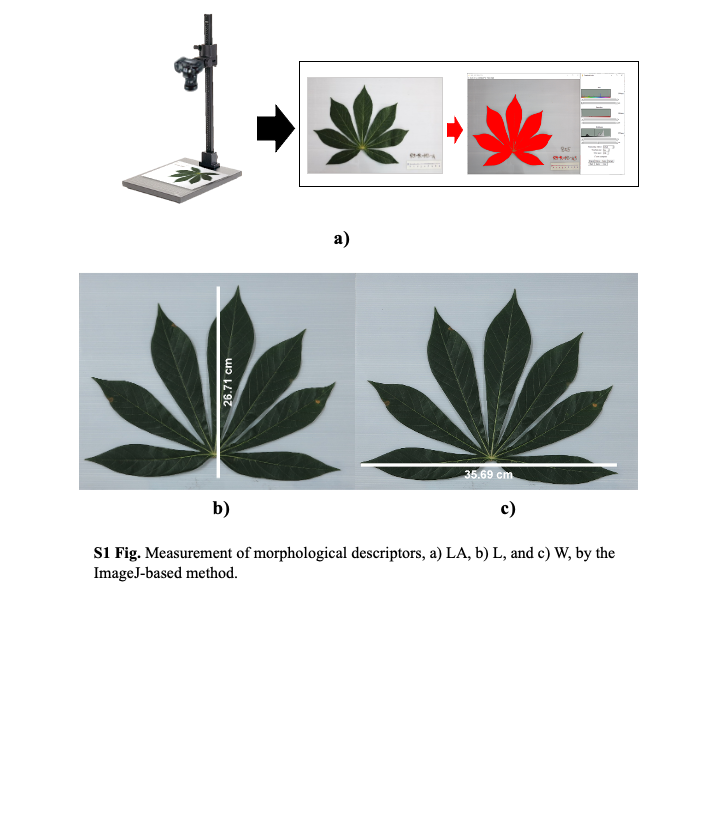

Supplement: S1 Fig — (TIFF) [file pone.0287293.s001.tiff]

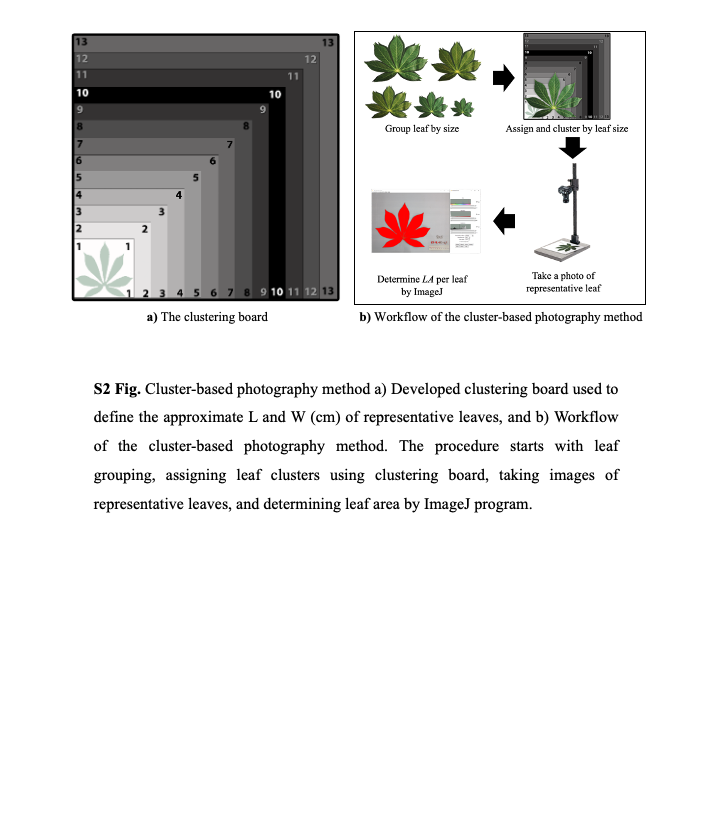

Supplement: S2 Fig — Cluster-based photography method a) Developed clustering board used to define the approximate L and W (cm) of representative leaves, and b) Workflow of the cluster-based photography method. The procedure starts with leaf grouping, assigning leaf clusters using clustering board, taking images of representative leaves, and determining leaf area by ImageJ program. (TIFF) [file pone.0287293.s002.tiff]

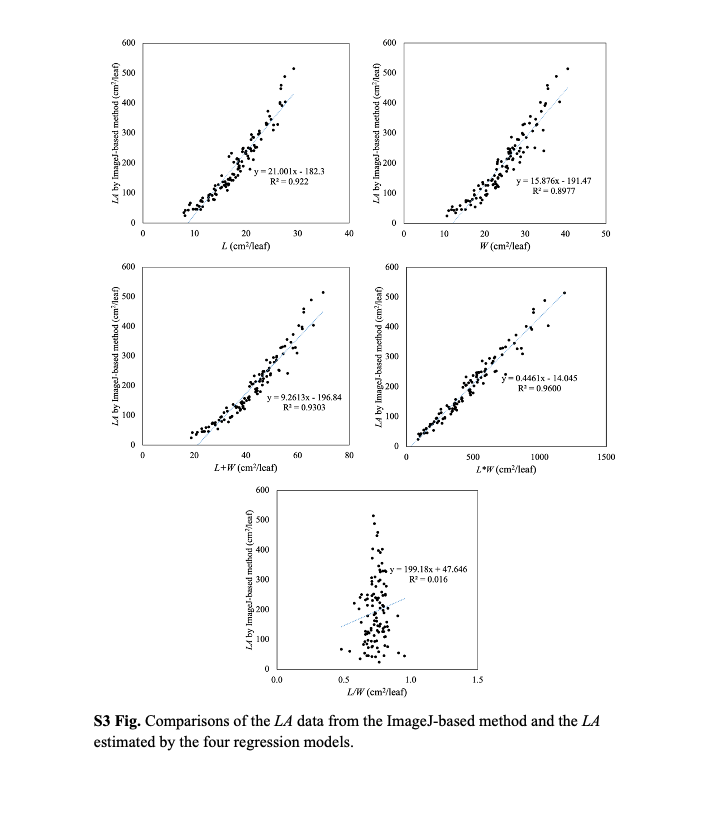

Supplement: S3 Fig — (TIFF) [file pone.0287293.s003.tiff]

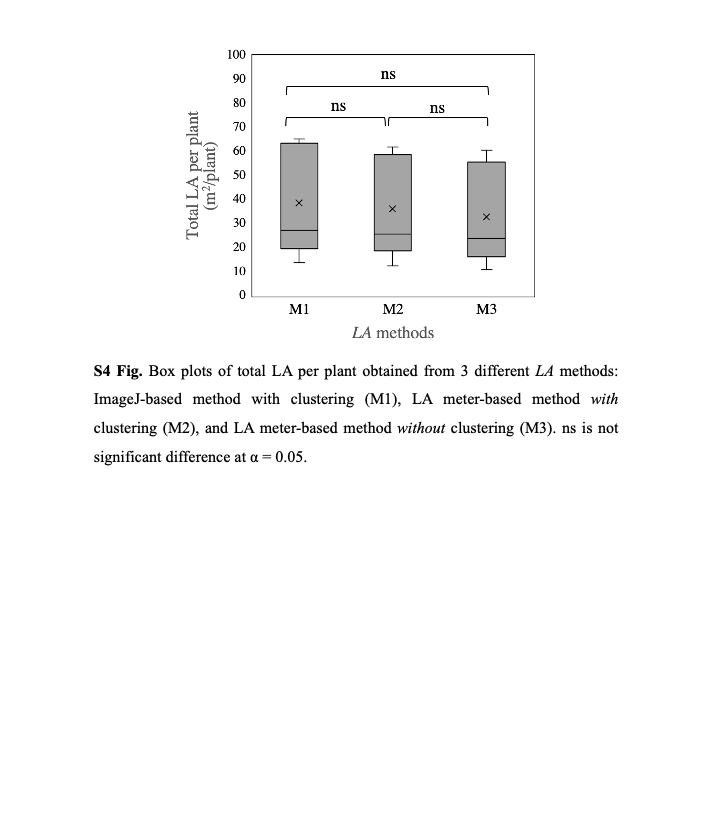

Supplement: S4 Fig — ns is not significant difference at α = 0.05. (TIFF) [file pone.0287293.s004.tiff]

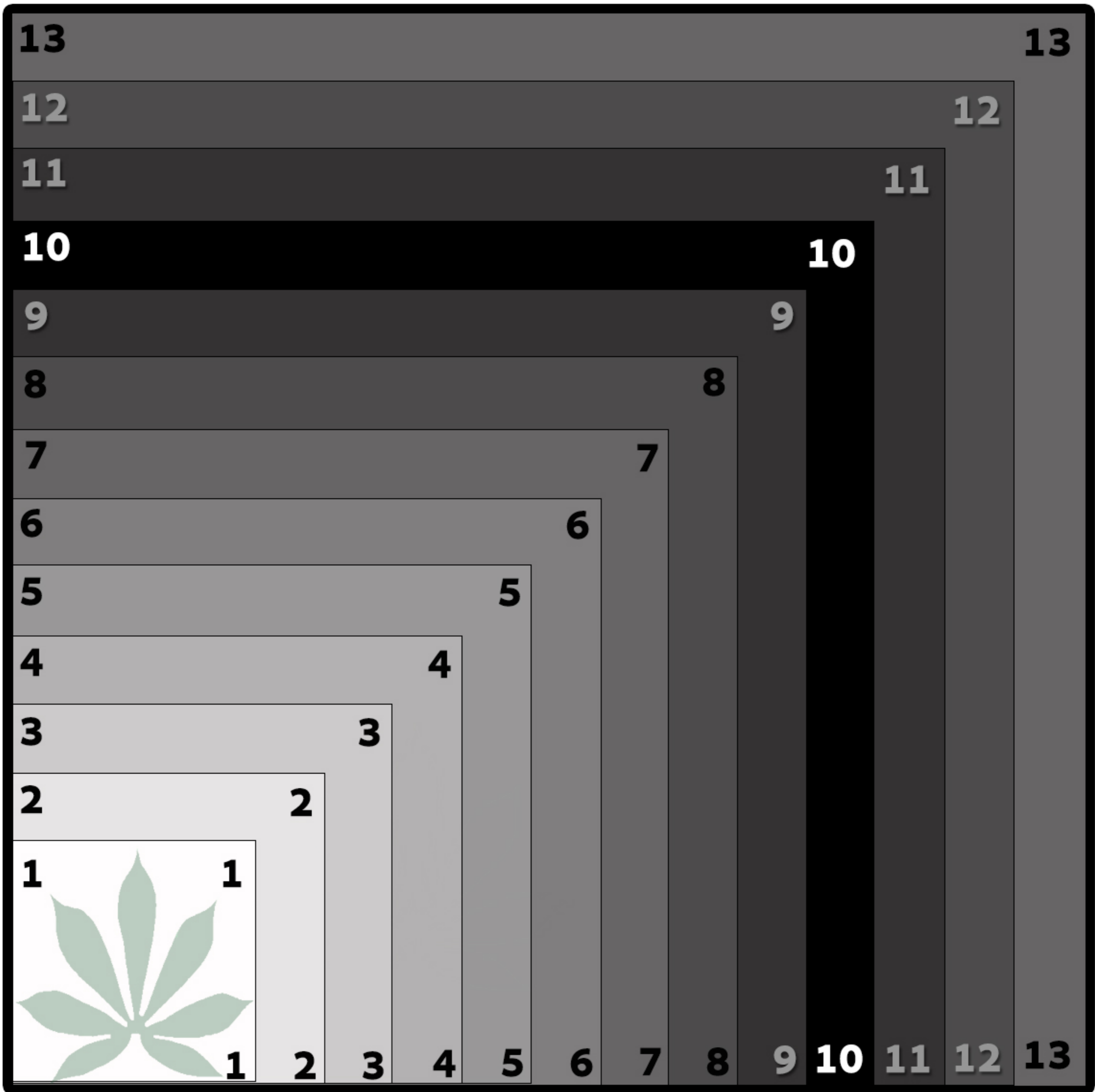

Supplement: S2 File — (PDF) [file pone.0287293.s006.pdf]
