# Supplementary material for: Cluster-based photography and modeling integrated method for an efficient measurement of cassava leaf area
Source: PLoS One. 2023 Oct 20;18(10):e0287293. doi: 10.1371/journal.pone.0287293 (PMC10588870; doi:10.1371/journal.pone.0287293)
Supplement: S3 File — (DOCX) [file pone.0287293.s007.docx]

**Supplementary information S3**

**Plant materials for simple regression model**

***Leaf morphology of 3 cultivars***

There are 3 cultivars used in this study, Kasetsart 50 (KU50), Rayong 9 (R9), and Hanatee (HN). These cultivars are selected because they are popular in the flour and food industry in Thailand. Regarding the 3 selected cassava varieties, they are good representatives of lanceolate-based morphological leaf traits, which is usually found in high yield varieties. Lobe shapes of these varieties (Fig. S3.1) could explain morphological characters of cassava leaves from ovoid to lanceolate (Fukuda et al., 2010). The leaf morphology of KU50 and R9 are similar, while HN is slightly different. The leaflet center of HN showed ovoid shape, whereas KU50 and R9 were lanceolate shape. The proposed method of *LA* estimators aims to provide a simple but effective measurement of cassava leaf area by taking into account variation in leaf morphology and size by genetic variety, growth and developmental stage of plants, and cultivation condition. For 1,899 leaf varieties obtained from 3 cassava genotypes (KU50, R9, and HN), 5 cultivation conditions (based on irrigation patterns and growth systems) covered, at least, variation of leaf length from 6.28 to 29.26 cm (average 16.6 ± 3.10 cm), leaf width from 5.00 to 40.58 cm (average 22.63 ± 4.71 cm), and leaf area from 11.73 to 514.89 cm^2^ (average 171.36 ± 63.16 cm^2^) (Table 2 in main manuscript). Corresponding to Phoncharoen and colleague’s work (2022), the variation of 1,899 leaves showed closely to the range of leaf length and leaf area in previous work that studied the leaf traits of 2,976 cassava leaves were randomly collected from 62 genotypes from 3 to 10 MAP, 3 positions (top, middle, and bottom) (Phoncharoen et al., 2022). The results of previous work showed that leaf length is 5.90-27.00 cm, leaf area is 14.09-470.98 cm^2^ confirmed that variation of leaf samples was sufficient for model development.

***Sample selection for simple regression model and model verification***

All 189 representative leaves were divided into 2 groups; 111 representative leaves used for simple linear regression model development and 78 representative leaves used for model testing. To generalize model, we needed the input data with a high variation to be good representative cassava leaves. Therefore, leaf samples were selected from 1-2 of 20 plants per plot grown in the field between 8-12 MAP because these ages included the high variation of leaf morphology. Each plot was grown with separate species and age. The distance between the plants and the row was 0.8 m × 1 m. The 111 leaves for create the simple regression model was obtained after clustering of 9 plants (total number of leaves is 1,821) and used to be representative of all leaves. Since the 111 leaves were obtained after clustering that were around 10% of total leaves, it could represent the diversity of all leaves.

The method was verified using the 78 representative leaves of KU50 and HN at 2 MAP grown under greenhouse (GH) were used for model verification as to generalize the approach to boarder *LA* measurements. The accuracy and precision assessed by using leaves from the 2 months-old GH plants, which had different in morphology and size by cultivars, leaf shapes, and developmental stage (Fig. S3.1), ensured the effectiveness of the method for general use.

***Implementation of hybrid method in a field***

For measuring total leaf area in a field by our hybrid method, we provided the clustering board and datasheet of S1.3 file for print and be used to measure the size of the leave, count, and record the number of leaves per cluster without detaching the leaves from the plant. After data collection, cluster identifier and number of leaves per cluster are filled in supplementary file S1- sheet S1.1, the table will give a value of total *LA* per plant.

In case of the morphology of palmate leaves differing from our cultivars; KU50, R9, and HN, our proposed method by design is easy to adapt and improve precision for all kinds of leaf morphology using the tools provided in supplementary file S1 Sheet-S1.2 for finding new regression model combined with scoring board. Noted that, the new measured *LA* is needed to determine a new regression model.

| 1. **Selected leaves from plants grown in field** | |
| --- | --- |
| **KU50** | **R9** |
| **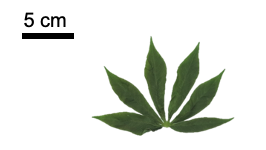** | **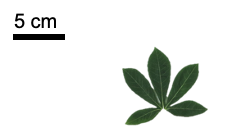** |
| **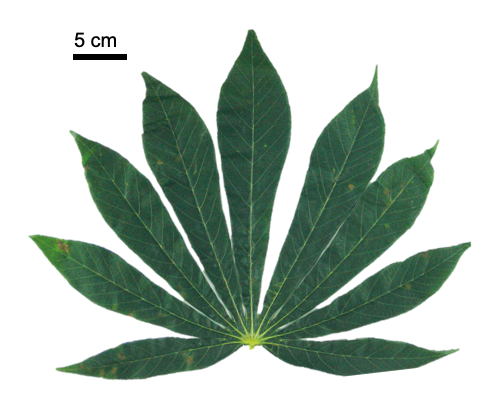** | **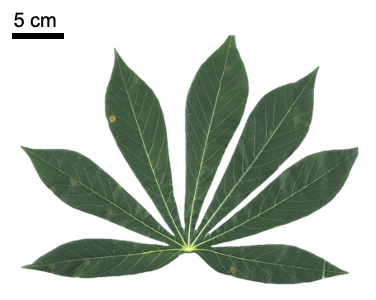** |

| 1. **Selected leaves from plants grown under greenhouse** | |
| --- | --- |
| **KU50** | **HN** |
| **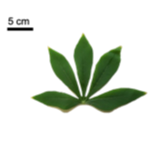** | **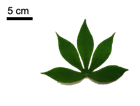** |
| **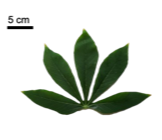** | **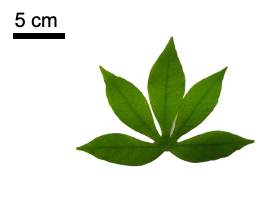** |

**Fig S3.1** Examples of selected leaves from plants grown in field (a) and under greenhouse (b)

**References:**

Phoncharoen P, Banterng P, Vorasoot N, Jogloy S, Theerakulpisut P. Determination of Cassava Leaf Area for Breeding Programs. Agronomy. 2022 Nov 29;12(12):3013.

Fukuda, W. M. G., Guevara, C. L., Kawuki, R., & Ferguson, M. E. (2010). *Selected morphological and agronomic descriptors for the characterization of cassava*. IITA.
